# Enhancing the mechanical and thermal properties of polypropylene composite by encapsulating styrene acrylonitrile with ammonium polyphosphate

**DOI:** 10.1186/s13065-019-0534-6

**Published:** 2019-01-30

**Authors:** Yi-jun Liao, Xiao-li Wu, Xin Peng, Zheng Zhou, Ju-zhen Wu, Fang Wu, Tao Jiang, Jia-xuan Chen, Lin Zhu, Tao Yi

**Affiliations:** 1 School of Materials Engineering, Chengdu Technological University, Chengdu, 611730 China; 2 Center of Big Data for Smart Environmental Protection, Chengdu Technological University, Chengdu, 611730 China; 3 Institute of Biopharmaceutical Technology, Zhejiang Pharmaceutical College, Zhejiang, 315100 China; 4 College of Architectural and Environmental Engineering, Chengdu Technological University, Chengdu, 611730 China; 5 National Engineering Research Center for Biomaterials, Sichuan University, Chengdu, People’s Republic of China; 6 College of Chemistry, Leshan Normal University, Leshan, 614004 China; 7 School of Chinese Medicine, Hong Kong Baptist University, Hong Kong, Special Administrative Region China

**Keywords:** Polypropylene, SAN–TAPP, Mechanical properties, Flame retardancy

## Abstract

**Backgrounds:**

In recent decades, incorporating polypropylene (PP) within flame retardants has proved to be an effective method of improving the thermal stabilities of PP, but too much adversely affects the mechanical properties of this polymer materials. Herein we report a novel multifunctional flame retardant, (styrene acrylonitrile)–(titanate-modified ammonium polyphosphate) (SAN–TAPP), to simultaneously improve the mechanical properties and thermal stability of PP composites.

**Methods:**

SAN–TAPP was synthesized by encapsulating SAN resins with functional titanate-modified APP (TAPP) and subsequently was incorporated into PP by a melt-blending process. The phase characteristics and morphology of SAN–TAPP were investigated, and the mechanical properties and thermal stability of different content of PP/SAN–TAPP composites were studied.

**Results:**

The results showed that the TAPP was almost entirely wrapped in the SAN resins and PP/SAN–TAPP composites exhibited the sea-island morphology. For the mechanical properties, the impact strength of PP/SAN–TAPP composite was significantly improved, especially 15 wt% SAN–TAPP filled PP/SAN–TAPP composite exhibiting 2.17 times higher than that of pure PP. And the tensile strength and modulus also increased by addition of SAN–TAPP. For the thermal stabilities, melting temperatures (T_m_) and residual char yield were improved. Furthermore, the LOI value of PP/SAN–TAPP composites increased from 19.8 to 27.5%; The 15 and 20 wt% SAN–TAPP filled in PP/SAN–TAPP composites passed the V-2 test of UL-94, and exerted the similar effect on the flame retardancy to TAPP with the same loading.

**Conclusions:**

These results revealed that a novel PP/SAN–TAPP composites with synthetically enhancement on the mechanical properties, thermal stabilities and flame retardancy, suggesting a strong correlation between the phase structure, mechanical and thermal properties.

## Backgrounds

Polypropylene (PP) has been widely used in the past decades due to its good mechanical properties, resistance to chemical agents, and excellent electrical insulation. Nevertheless, several critics such as low impact resistance, flammability and low thermal stabilities restrict its applications [1–4]. Improving its impact strength and thermal properties, has increasingly attracted the attention of many researchers. In recent decades, incorporating functional nanoparticles into PP has proved to be an effective method for improving thermal property of PP. However, a high content of nanoparticles may lead to the reduction of the mechanical properties, especially the elastic modulus, tensile strength and high-temperature creep deformation [5–8].

Blending PP with rigid polymers to synthesize a binary or ternary system is a traditional method to improve mechanical and thermal properties simultaneously [9–14]. Recently, rigid polymers of nylon-6 [15, 16], polymethyl methacrylate [17, 18], acrylonitrile–butadiene–styrene (ABS) [19–22] and styrene–acrylonitrile (SAN) [23] have been frequently reported. SAN copolymer plays an important role in many industries owing to its high weathering ability [24]. Kim et al. [25] demonstrated that PC/SAN had developed useful mechanical properties. Yu et al. claimed that SAN can improve impact strength of isotactic polypropylene (*i*PP) [26]. Also, other researches have explored the use of SAN as a reinforcing agent in polymer material [27].

The high flammability of PP limits its applications, thus improving the fire retardancy of PP is the focus of many researches. In recent decades, adding flame retardants (FRs) into polymer materials is well known the main approaches. As a member of polymeric flame retardant additives, ammonium polyphosphate (APP), has received great attention due to its synergistic effect between phosphorus (P) and nitrogen (N), and highly effective catalyzing carbonization effect to promote the char formation. Besides, APP is as an intumescent flame retardant (IFR) with unmatched halogen-free, low-smoke and low-toxicity. However, like many other flame retardants additives, high APP content in a polymer (such as 20 wt%) results in the deterioration of its mechanical properties due to the thermodynamic incompatibility between APP and the polymer matrix [28–30]. Very recently, to overcome this problem, many researchers have covalently grafted polymeric flame retardant groups onto the polymer matrix or have modified the flame retardant with functional groups. For instance, Wang et al. wrapped ammonium polyphosphate with melamine-containing polyphosphazene (PZMA@APP) to improve flame retardancy and mechanical performance of EP composites [31]. Shao et al. modified APP via an ion exchange reaction with ethylene diamine, and obtained a novel flame retardant of polypropylene [32]. While modified APP has high flame retardancy, its low cross-linking would result in the deterioration of physical properties and thermal stabilities; this remains a problem for phosphorus-containing flame retardants. Therefore, it is necessary to further modify the phosphorus-containing flame retardant system to enhance both the thermal stabilities and mechanical properties of PP.

In this study, APP modified with titanate coupling agent (TAPP) was wrapped with SAN to produce a multifunctional flame retardant, SAN–TAPP; subsequently SAN–TAPP was incorporated into PP to obtain PP/SAN–TAPP composites. In this system, SAN is utilized to enhance the mechanical strength as a rigid body, and most importantly, it is expected to exert synergistic effect with TAPP to improve thermal properties and flame resistance of PP. Treating APP with titanate coupling agent aims to modify the interface between SAN and APP. For comparison, PP/(SAN + TAPP) was prepared by a one-step melt-blending process and PP/TAPP was also synthesized. The mechanical and thermal properties and flame retardancy of all three (PP, PP/TAPP,  and PP/SAN–TAPP composites) were characterized by impact and tensile testing, thermos gravimetric analysis (TGA), differential scanning calorimetry (DSC), scanning electron microscopy (SEM), X-ray diffraction (XRD) and flammability properties.

## Methods

### Materials

Polypropylene (PP, MFI = 27 g/10 min) was purchased from Kingfa Science and Technology. Co., Ltd (Guangzhou, China). SAN (HF-1095A) was purchased from Huafeng Corporation (Shenzheng, China). Chlorinated paraffin (CP) and Styrene maleic anhydride (SMA) were obtained from Shanghai Sunny New Technology Development (Shanghai, China). Titanate coupling agent (TCA) and APP were purchased from Tianchang hongsheng fine chemical Corporation (Shanghai, China). The starting compositions of the respective blends are presented in Table 1. All materials used in the blends were first dried at 80 °C and then accurately weighed.

**Table 1:** The composition of pure PP, PP/TAPP20 and PP/SAN–TAPP composites

Samples	PP (wt%)	SAN–TAPP (wt%)	TAPP (wt%)	SMA (wt%)	CP (wt%)
PP	100	0	0	2	0.5
PP/SAN–TAPP5	100	5	0	2	0.5
PP/SAN–TAPP10	100	10	0	2	0.5
PP/SAN–TAPP15	100	15	0	2	0.5
PP/SAN–TAPP20	100	20	0	2	0.5
PP/(SAN10 + TAPP10)	100	20	0	2	0.5
PP/TAPP20	100	0	20	2	0.5

### Synthesis of TAPP

Initially, APP was added into ethanol in a weight ratio of 1:3 with stirring. 10 wt% titanate coupling agent relative to APP/ethanol mixture was added dropwise. The mixture was magnetically stirred for 40 min at 50°C and then heated at 80 °C to remove all water. The dried samples were grounded for characterization and were grafted onto SAN.

### Synthesis of SAN–TAPP

SAN–TAPP was prepared by melt-blending SAN with TAPP. Initially, pure SAN and TAPP at the weight ratio of 1:1 were premixed in a high speed mixer (SHR-10A, Coperion Heng AO Machinery, Nanjing, China). Then the mixtures were fed into a twin screw co-rotating extruder (SHJ-36, Coperion Heng AO Machinery, Nanjing, China) with L/D 40 operating at a speed of 30 rpm/min. Compounding was carried out at 165, 175, 180, 185, 190, 195 and 190 °C in sequential heating zones. It was then cooled, cut, and finally dried at 90 °C for 8 h to remove all water.

### Preparation of PP/SAN–TAPP composites

PP/SAN–TAPP composites with different loading of SAN–TAPP were also synthesized by a melt-mixing process. Pure PP, SAN–TAPP, SMA and CP were mixed, melt-blended, cooled, cut, and then dried. The processing temperatures were set as 160, 180, 190, 200, 200, 200, 200, 210, and 210 °C and the screw rotating speed was 30 rpm/min.

**Fig. 1:**
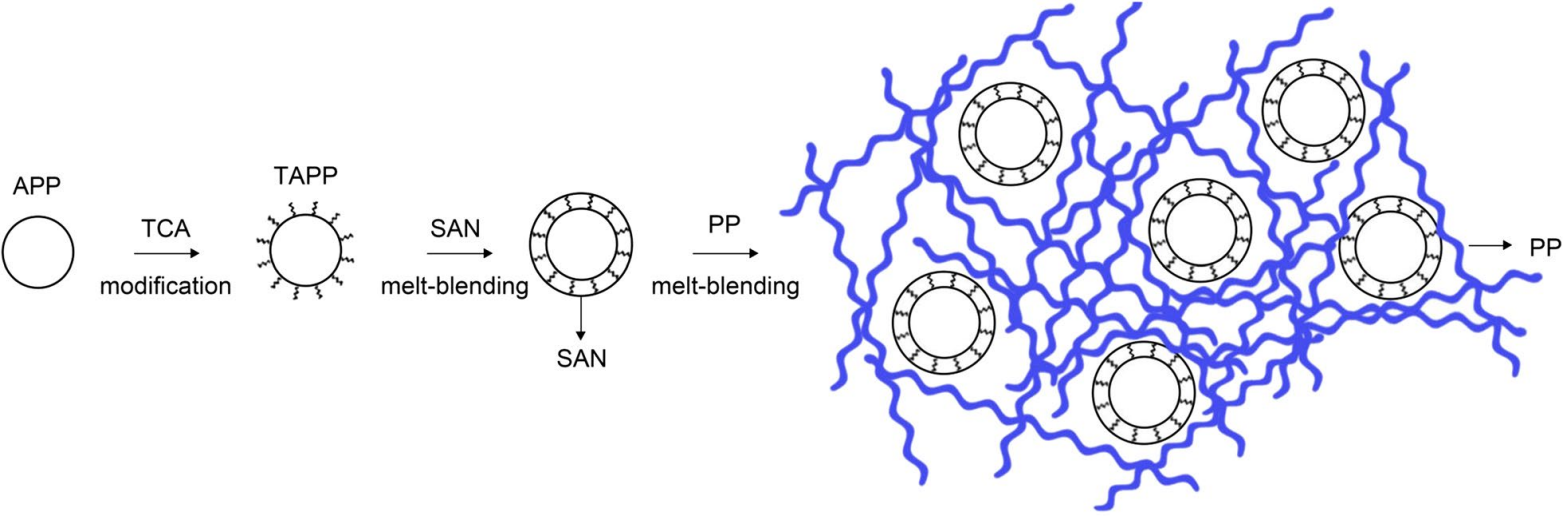
The preparation process and mechanism of SAN–TAPP and PP/SAN–TAPP composites

PP/TAPP20 composite with 20 wt% TAPP was prepared for comparison. Moreover, to study the influence of the melt process on the properties of the composites, 10 wt% SAN and 10 wt% TAPP filled PP composite was also prepared by one step melt-blending. The process is as follow: for this process, the pre-mixtures of pure PP, SAN, TAPP, SMA and CP were all fed into the extruder at the same temperatures and rotating speed as described above for the PP/SAN–TAPP composites. The whole preparing process and mechanism are displayed in Fig. 1.

Some extrudates were immediately molded by an injection molding machine (TC-150-P, Tiancheng Machinery Co. Ltd., China) at 180, 195, and 205 °C in sequential zones from hopper to mold to obtain specific sheets (dog bone-shaped specimens (150 mm × 10 mm × 4 mm) and rectangular samples (80 mm × 10 mm × 4 mm)) for mechanical and thermal testing and morphological examination.

### Material characterization

The phase constituents of TAPP, SAN–TAPP, PP/TAPP20 and PP/SAN–TAPP composites were evaluated using an X-ray diffractometer (XRD, Philips PC-APD) with a CuKα (40 mA and 40 kV) radiation source of 0.154 nm wavelength at room temperature of 25 °C. The functional groups were examined using a Fourier transform infrared spectroscope (FTIR, Nicolet, 170SX, Wisconsin, USA) in the wave number range of 400 to 4000 cm^−1^ by pressing the samples and KBr into a membrane.

### Mechanical properties testing

Measurements of tensile properties of PP/SAN–TAPP composites were carried out on a universal testing machine (WDW-100, Tianjin Meites Testing machine factory, China) using dog bone-shaped specimens (150 mm × 10 mm × 4 mm) according to the standard of GB/T 1040.2-2006 at room temperature. The assay was performed under a liner deformation loading rate of 50 mm/min until mechanical failure occurred. Three replicates were performed for each measurement. The impact strength was assessed on a beam impact testing machine (XJJ-5, Chengde Shipeng Testing Machine Co. LTD, China) at ambient temperature using rectangular samples (80 mm × 10 mm × 4 mm) in terms of GB/T 1043.1-2008 standard. For each measurement, three specimens were used.

### Morphological observations

The morphologies of SAN–TAPP, pure PP and PP/SAN–TAPP composites containing 10 and 20 wt% SAN–TAPP were characterized by scanning electron microscopy (SEM, S-900, Hitachi, Japan) at magnifications of 2000×, operating at an accelerating voltage of 5 kV. The specimens were cryogenically fractured in liquid nitrogen, and the fracture surfaces were coated with platinum to a depth of 10 Å.

### Thermal deformation behavior and viscosity analysis

The thermal properties of the composites were determined using a differential scanning calorimeter (DSC, Q2000, TA instruments Inc., USA). Samples were subjected to a stream of pure nitrogen flowing at a rate of 50 ml/min and heated at 10 °C/min from 25 to 220 °C. Thermogravimetric analysis (TGA) measurements were carried out with a thermal analyzer (Q5000, TA instruments Inc., USA) from 30 to 700 °C at a heating rate of 10 °C/min under N_2_ atmosphere.

The heat deflection temperature (HDT) and vicat softening temperature (VST) of pure PP, PP/TAPP20 and PP/SAN–TAPP composites were assessed using a thermal deformation and vicat softening temperature tester (XWB-300B, Chengde Shipeng Testing Machine co. LTD, China) with silicone oil as warming medium. To test HDT values, rectangular samples (80 mm × 10 mm × 4 mm) were scanned from 25 °C to deformation temperature at a heating rate of 120 °C/h under a perpendicular loading weight of 75 g (bending normal stress: 0.45 MPa) in line with GB/T1634.2-2004. The VST values of all specimens were measured under a loading weight of 1000 g, heating from 25 °C to vicat softening temperature at a rate of 50 °C/h in terms of GB/T 1633-2000. The flame-retardant performance was characterized by vertical burning test (UL-94) and limiting oxygen index (LOI). Vertical burning ratings of these samples were determined using a CZF-5 instrument (Nanjing Qionglei Instrument Co., China) with a sample size of 125 mm × 12.5 mm × 3 mm according to ISO 1210-1992. Limiting oxygen indexes (LOI) of all samples (130 mm × 6.5 mm × 3 mm) were determined on a JF-3 oxygen index meter (Nanjing Jiangning Analysis Instrument Co., China) according to ASTM D2863-2012 standard.

## Results and discussion

### Characterization of SAN–TAPP

**Fig. 2:**
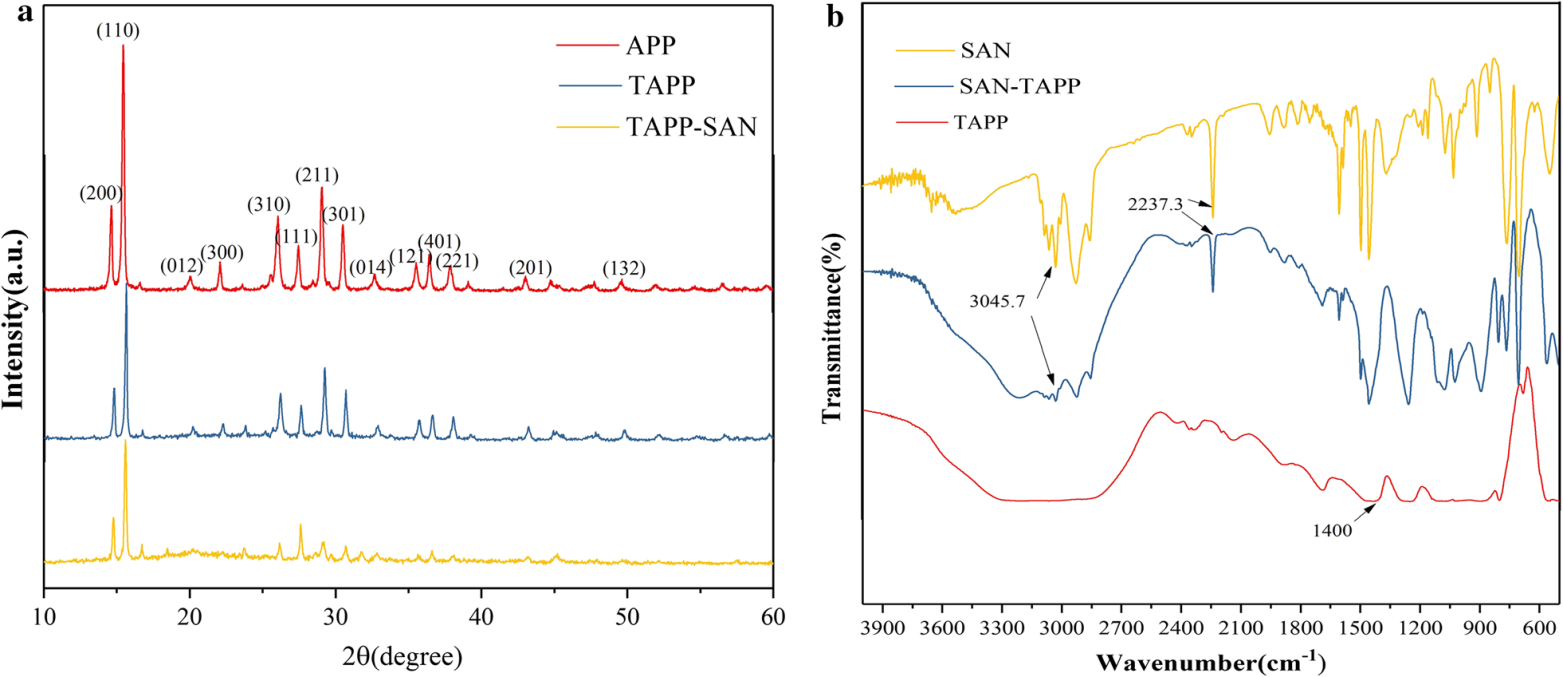
**a** XRD patten; **b** FTIR spectra of APP, TAPP and SAN–TAPP

The XRD patterns of APP, TAPP and SAN–TAPP are displayed in Fig. 2a. For TAPP sample, crystal peaks could be clearly observed at 2θ values of around 14.7°, 15.5°, 26.1°, 27.5°, 29.1°, 30.5° and 36.4°, which were consistent with (200), (110), (310), (111), (211), (301) and (401) planes of APP II, respectively [33]. TAPP showed similar diffraction angles of 2θ, with lower peak intensity as compared to APP, which was ascribed to the long chain of the titanate group modifying APP. A similar pattern was also observed for the SAN–TAPP sample, which had the lowest peak intensity among these samples. The low intensity are attributed to the amorphous phase of SAN encapsulating the surface of APP.

**Fig. 3:**
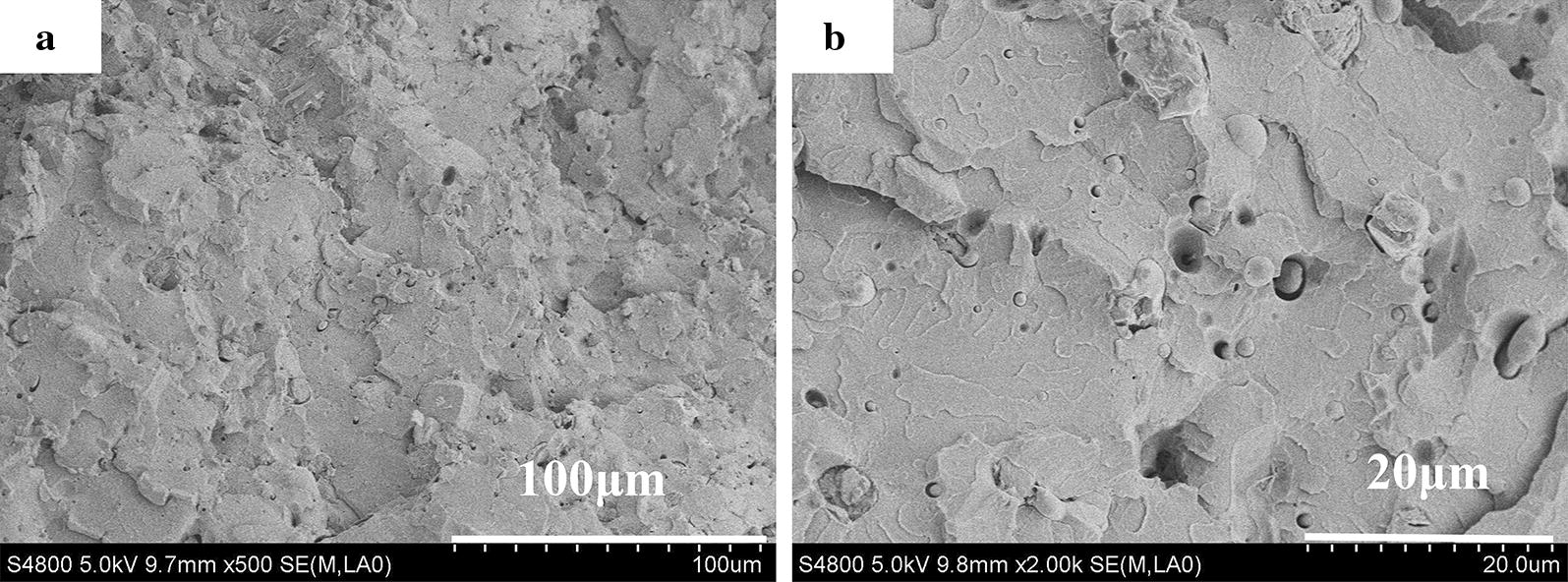
SEM images of SAN–TAPP **a** ×500; **b** ×2000

The Fig. 2b shows the FTIR spectra of TAPP and SAN–TAPP. The characteristic peaks of APP were observed for all specimens. The FTIR spectrum of SAN–TAPP exhibited some additional peaks, such as the peaks around 2237.3 cm^−1^ and 3028.0 cm^−1^ that corresponded to, respectively, C≡N stretching vibrations in acrylonitrile and C–H stretching vibrations of benzene in styrene coming from SAN [34, 35]. The morphology of SAN-g-TAPP’s fracture surface is shown in Fig. 3. It could be seen that the surface of SAN–TAPP showed no wrinkle with only some nano-spheres found there. This result confirmed the brittleness of SAN–TAPP and further proved that most TAPP particles were wrapped in SAN copolymer; in other words SAN-APP was successfully synthesized.

### XRD analysis of PP/SAN–TAPP composites

**Fig. 4:**
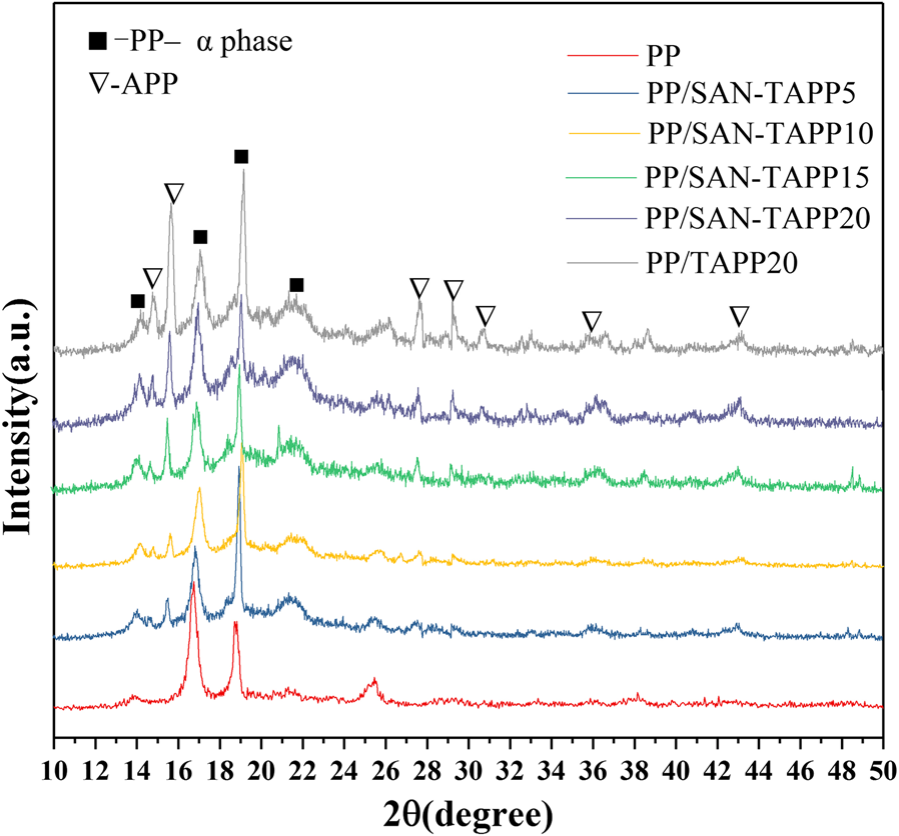
XRD patterns of pure PP, PP/TAPP20 and PP/SAN–TAPP composites

PP is known to be a polymorphous crystal, and is consist of three crystalline forms designated as monoclinicα-phase, trigonal β-phase, and orthorhombic γ-phase. α-phase dominates; β-phase and γ-phase are induced when nucleating agents are added into the PP matrix [20–22]. The XRD patterns of pure PP, PP/TAPP20 and PP/SAN–TAPP composites are displayed in Fig. 4. It could be seen that all specimens had crystal peaks at 2θ values of around 13.9°, 16.8°, 18.8° and 21.2°, which were the typical diffraction peaks of the monoclinic α-phase of PP crystals. These peaks corresponded to (110), (040), (130) and (131) planes, respectively [36]. The diffraction peaks of APP also appeared in PP/TAPP20 and PP/SAN–TAPP composites, the intensity of these peaks increased with the increase of TAPP content. Peaks corresponding to the β and γ-crystalline phases of PP crystal were not observed, which indicates that SAN and TAPP have no obvious effect on the crystallization behavior of PP.

### FTIR analysis of PP/SAN–TAPP composites

**Fig. 5:**
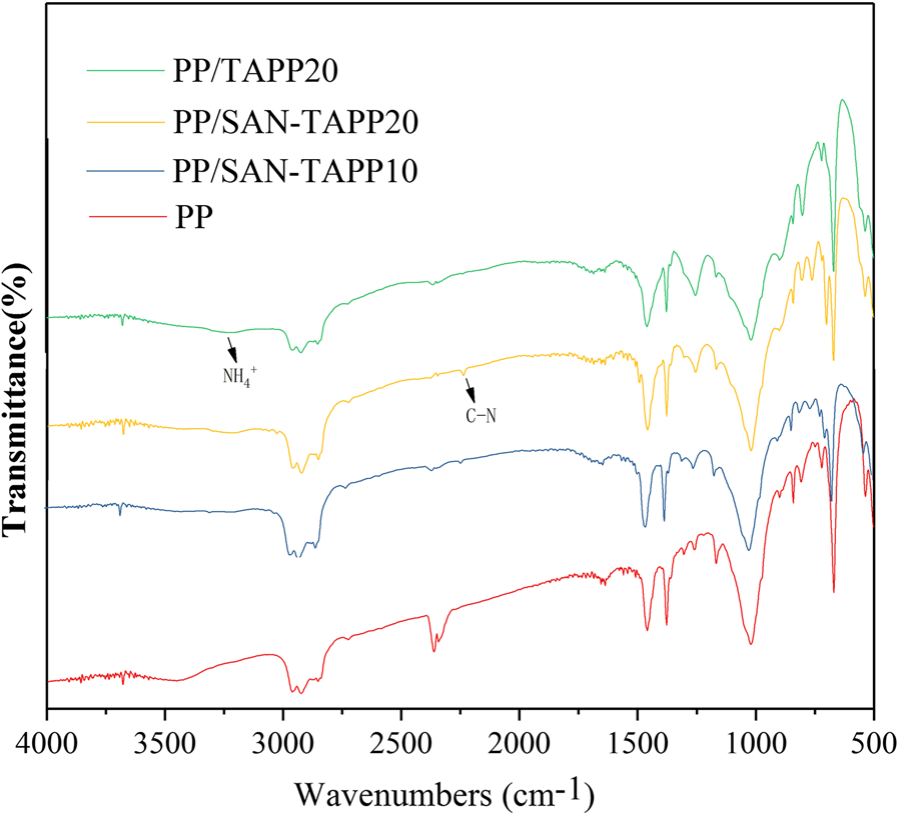
FTIR spectra of pure PP, PP/TAPP20 and PP/SAN–TAPP composites

Figure 5 shows the FTIR spectra of pure PP, PP/TAPP20 and PP/SAN–TAPP composites. The FTIR spectra of all specimens exhibited characteristic peaks of PP phase, with the absorption peaks of around 1458.5 cm^−1^ and 1377.2 cm^−1^ being consistent with the CH_3_ or CH_2_ deformation vibration, respectively, and the peaks at 2918.4 cm^−1^ and 2854.2 cm^−1^ corresponding to stretching vibrations of CH_2_ [35, 37]. For PP/TAPP20, new absorption peaks appeared at around 3300–2960 cm^−1^, which were assigned to the NH_4_^+^ asymmetry stretching vibration of TAPP [38]. For PP/SAN–TAPP composites, typical peaks of TAPP were still present, and the characteristic peaks of SAN also appeared at around 2237.2 cm^−1^ and 3028.0 cm^−1^. However, the spectra of PP/SAN–TAPP composites showed no other additional absorption peaks compared to the spectra of pure PP and PP/TAPP20. This means that there was no chemical reaction between PP and SAN–TAPP, which suggests immiscibility between PP and SAN–TAPP [39].

#### Scanning electron microscopy

**Fig. 6:**
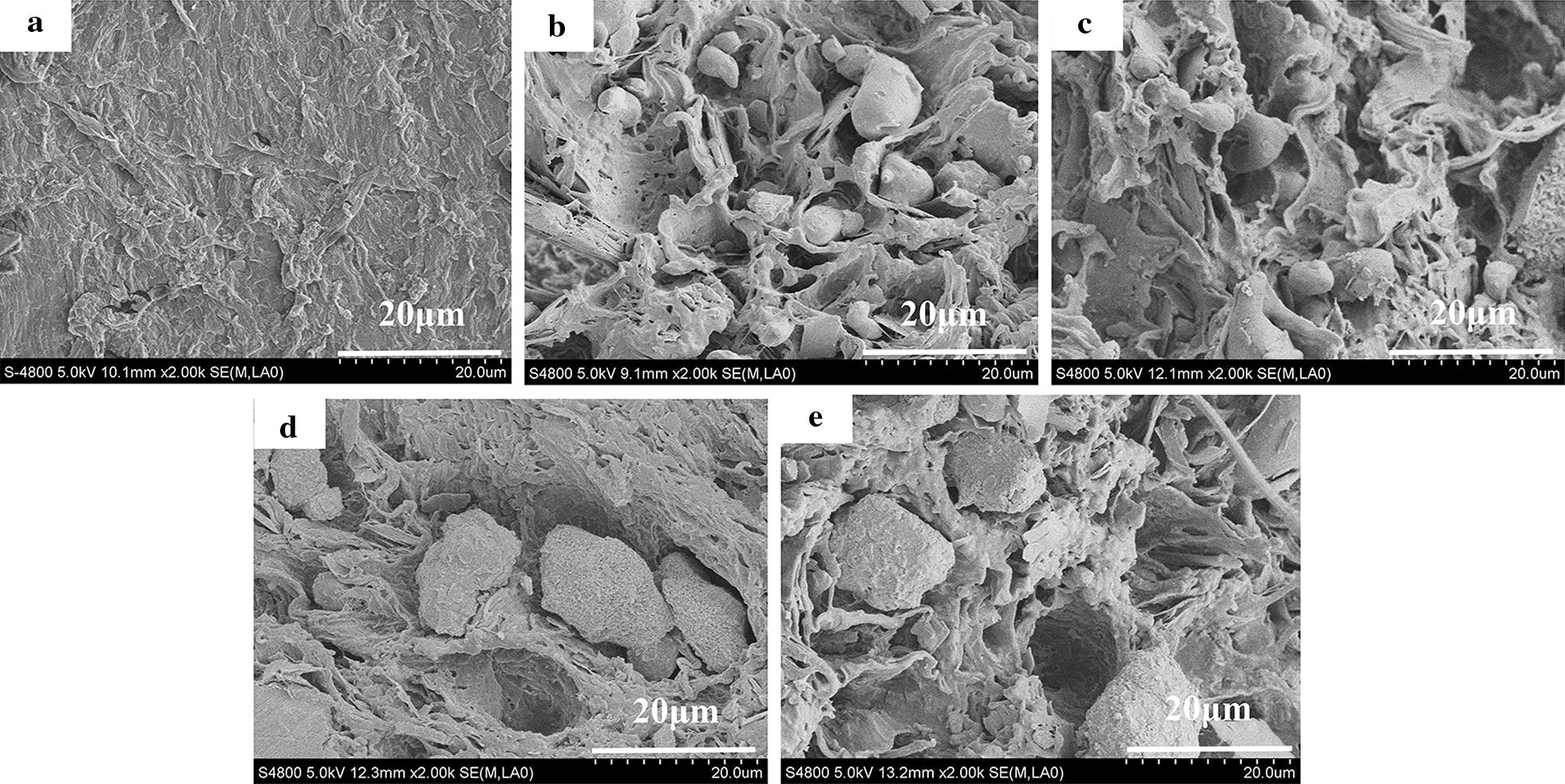
SEM morphologies of the fractured surface of **a** pure PP ×2000; **b** PP/TAPP20; **c** PP/(SAN10 + TAPP10) ×2000; **d** PP/SAN–TAPP10 ×2000; **e** PP/SAN–TAPP20 ×2000

In order to examine the phase compatibility and distribution of SAN–TAPP in the PP matrix, the morphologies of fracture surfaces of pure PP, PP/TAPP 20, 10 and 20 wt% SAN–TAPP filled PP/SAN–TAPP composite and 10 wt% SAN- and 10 wt% TAPP-filled PP/(SAN + TAPP) composite were investigated by SEM. As shown in Fig. 6, the fractured surface of pure PP was flat without stripes, suggesting a brittle material. For PP/TAPP20 composite (Fig. 6b), TAPP particles ranging from 1 to 14 μm were dispersed in the surface of PP, which revealed the immiscibility between TAPP and PP matrix. The 10 wt% SAN- and 10 wt% TAPP-filled PP/(SAN + TAPP) composite also exhibited TAPP particles on the surface (Fig. 6c). And some new like “fiddlehead” appeared which were consistent to the SAN copolymer, resulting from the one melt-blending process. For PP/SAN–TAPP composites, an irregular structure like sea-island was distinctly observed and many stripes were found there (Fig. 6d, e). The “island” were irregular spheres and the sphere’s morphologies varied with the content of SAN–TAPP. In 10 wt% SAN–TAPP filled PP/SAN–TAPP (Fig. 6d), the spheres were with size ranging from 12 to 20 μm. TAPP particles were not observed, which was likely due to the wrapping of SAN. Some cavities like meteor craters were found probably arising from the dissociation of SAN–TAPP microspheres from PP matrix during the material fractures [40]. In 20 wt% SAN–TAPP filled PP/SAN–TAPP composite (Fig. 6e), SAN–TAPP spheres were still present, and some TAPP particles existed which was possibly due to the aggregation of TAPP particles in SAN during the first melting process. Generally, 10 wt% SAN–TAPP filled PP/SAN–TAPP composite showed the most refine and homogeneous morphology among these composites.

### Mechanical properties

**Fig. 7:**
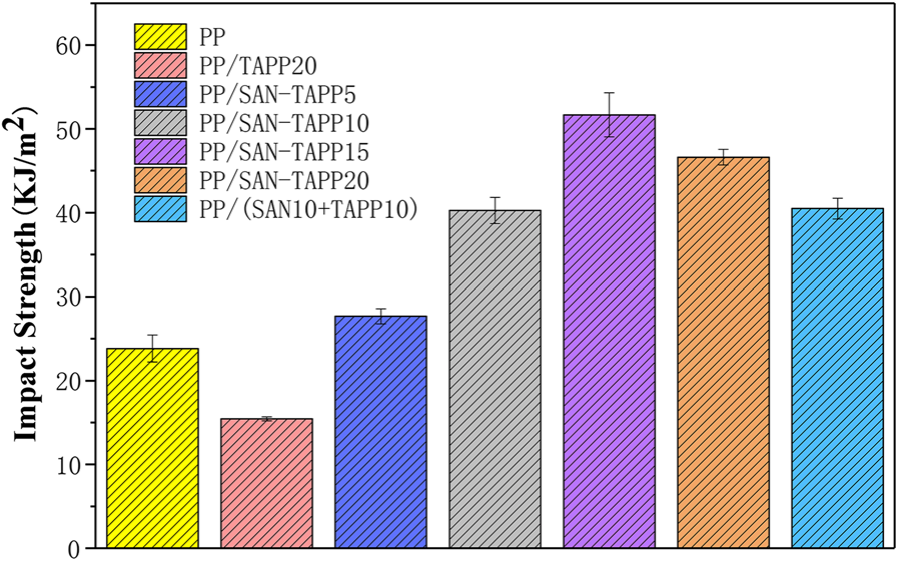
Impact strength of pure PP, PP/TAPP20 and PP/SAN–TAPP composites

Figure 7 displays the impact properties of pure PP, PP/TAPP20 and PP/SAN–TAPP composites. Pure PP showed an impact strength of 23.83 kJ/m^2^. 20 wt% TAPP filled PP/TAPP20 composite exhibited an impact strength of 15.45 kJ/m^2^, which was significantly lower than that of the pure PP. This suggests that high content of TAPP indeed leads to the deterioration impact properties of its polymer composite. The impact strength of 5 wt% SAN–TAPP filled PP/SAN–TAPP slightly increased compared to that of pure PP. When the content of SAN–TAPP rose to 15 wt%, the impact strength was improved to the maximum value of 51.68 kJ/m^2^, which was 2.17 times higher than that of pure PP. The 10 wt% SAN- and 10 wt% TAPP-filled PP/(SAN + TAPP) composite by one-step process had a tensile strength of 40.49 kJ/m^2^, which was lower than that of the 20 wt% SAN–TAPP filled PP/SAN–TAPP composite.

**Fig. 8:**
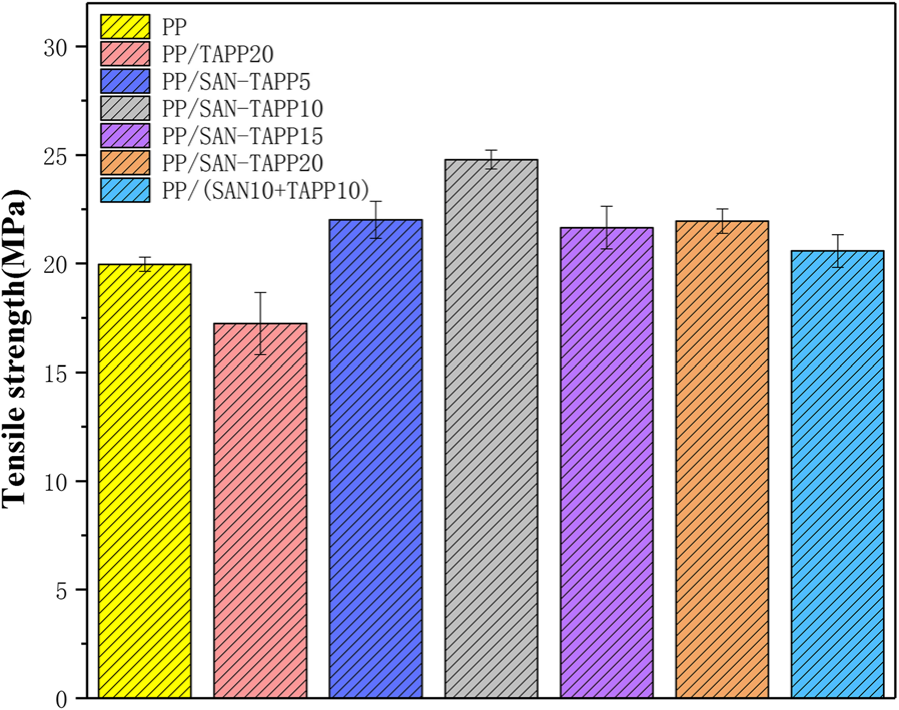
Tensile strength of pure PP, PP/TAPP20 and PP/SAN–TAPP composites

**Fig. 9:**
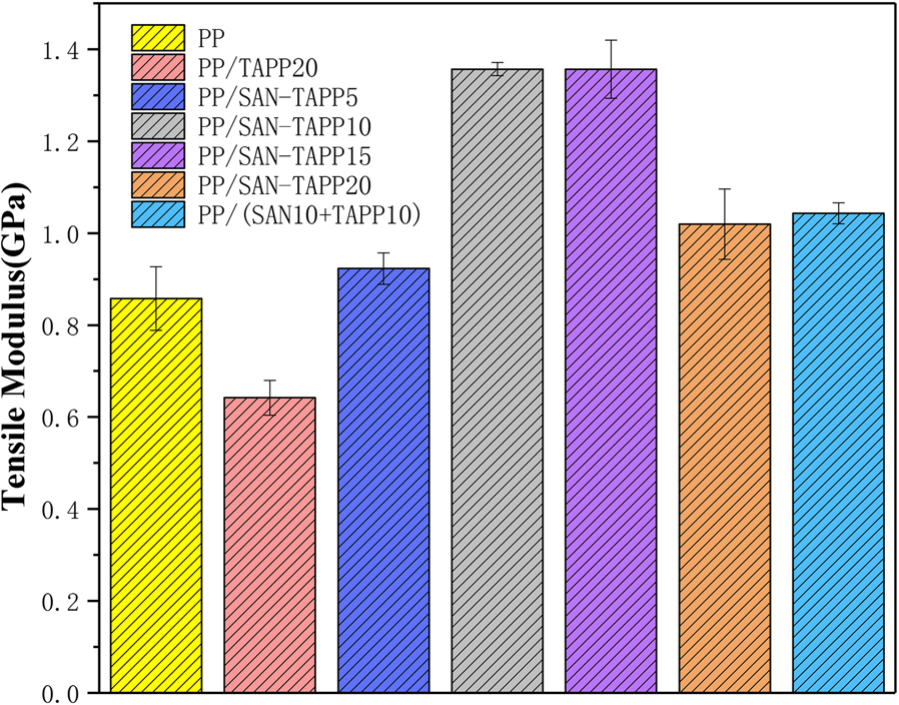
Tensile modulus of pure PP, PP/TAPP20 and PP/SAN–TAPP composites

Figures 8 and 9 show the tensile strength and tensile modulus of pure PP, PP/TAPP20 and PP/SAN–TAPP composites, respectively. The tensile strength exhibited a trend similar to that of impact strength, with a maximum value occurred in 10 wt% SAN–TAPP filled PP/SAN–TAPP composite. All PP/SAN–TAPP composites showed a slightly higher tensile strength than that of pure PP. The increase of tensile modulus was at maximum for 10 wt% SAN–TAPP filled PP/SAN–TAPP composite and 15 wt% SAN–TAPP filled PP/SAN–TAPP composite. The 10 wt% SAN- and 10 wt% TAPP-filled PP/(SAN + TAPP) composite by one-step process also had a lower tensile modulus as compared to 20 wt% SAN–TAPP filled PP/SAN–TAPP composite.

Impact property plays a critical role in engineering applications. The result demonstrates that the toughness was significantly enhanced by adding SAN–TAPP into the PP matrix, as a function of the content of SAN–TAPP, clearly indicating that SAN–TAPP can serve as a rigid reinforcer in PP matrix. The enhancement in impact strength of PP/SAN–TAPP composites was mostly attributed to the formation of a sea-island morphology. When PP/SAN–TAPP composites are subjected to impact loading, the “islands” were pulled out as the load transferring to, accompanied by void growth at the interface or cavitation of SAN–TAPP, and finally resulting in more energy absorption and effective resistance to crack propagation [20, 41]. The impact strength of 10 wt% SAN- and 10 wt% TAPP-filled PP/SAN–TAPP composite was lower than that of 20 wt% SAN–TAPP filled PP/SAN–TAPP composite, which could be attributed to the dispersion of more and larger TAPP particles in PP matrix. Moreover, 20 wt% SAN–TAPP filled PP/SAN–TAPP composite exhibited a more refined and homogeneous morphology in comparison to 10 wt% SAN- and 10 wt% TAPP-filled PP/SAN–TAPP composite. García et al. [2] confirmed that the large particles would create large voids that may destroy the structural integrity of a polymer matrix, ultimately resulting in specimen failure. Thus, smaller particles are more desirable. In addition, in 20 wt% SAN–TAPP filled PP/SAN–TAPP composite, the TAPP was wrapped in SAN such that SAN served as a shell and connected more tightly connect with the PP matrix, leading to higher resistance to separation when the composites were subjected to impact loading [19].

The reason for maximum reinforcement in tensile strength and tensile modulus at 10 wt% or 15 wt% SAN–TAPP filled PP/SAN–TAPP composite could be the dispersion of SAN–TAPP in the PP matrix. With respect to the distribution and the size of SAN–TAPP in PP matrix, 10 wt% and 15 wt% SAN–TAPP filled PP/SAN–TAPP composite had a more homogeneous morphology than that of other PP/SAN–TAPP composites. This decreased the stress-concentration points in the interfacial regions, and resulted in the reinforcement of tensile properties.

### Thermal properties of PP/SAN–TAPP composites

#### DSC analysis

**Fig. 10:**
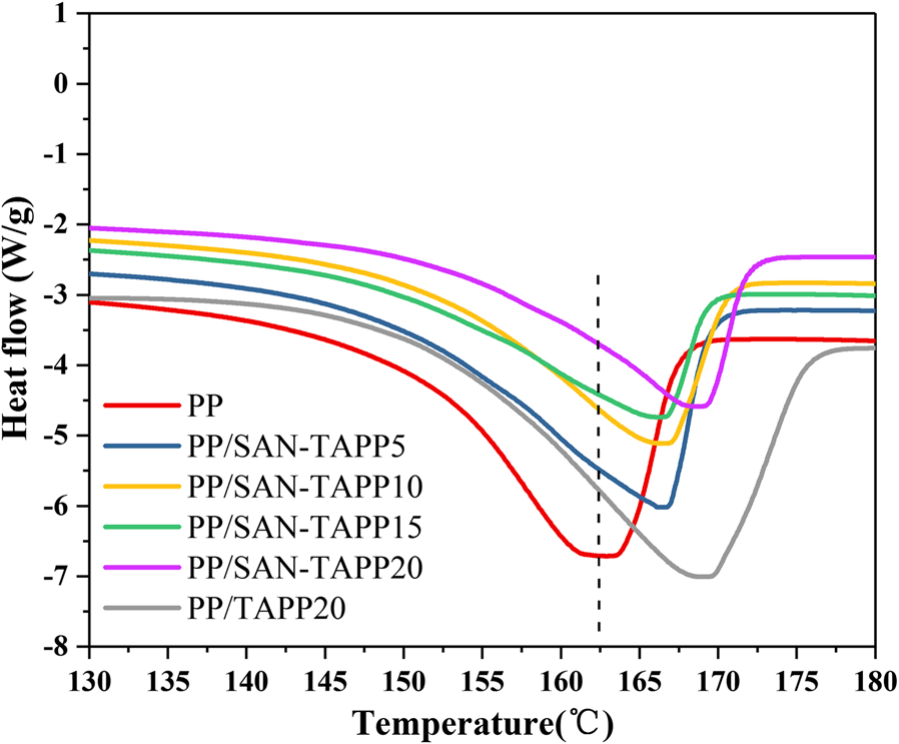
DSC patterns of pure PP, PP/TAPP20 and PP/SAN–TAPP composites

The melting endotherm of heating pure PP, PP/TAPP20 and PP/SAN–TAPP composites are displayed in Fig. 10. The melting temperatures (T_m_) of these specimens were clearly affected by the presence of TAPP and SAN. Pure PP exhibited an endothermic melting peak at about 163.3 °C. The 20 wt% TAPP filled PP/TAPP20 composites showed a largely higher T_m_ as compared to that of pure PP, which was due to the catalyzing carbonization effect of TAPP [31]. In fact, all PP/SAN–TAPP composites exhibited an increase in T_m_ as compared with the pure PP sample. In addition, the T_m_ increased as the SAN–TAPP content increased. When the content increased up to 20 wt%, the T_m_ reached its maximum value at 170 °C, which was close to that of 20 wt% TAPP filled PP/TAPP20 composites. It indicates that the presence of SAN–TAPP would enhance the thermal properties of PP/SAN–TAPP composites and exert the same effect to TAPP with the same loading. This is likely due to the synergistic reinforcement of TAPP and SAN and strong interfacial reaction (interaction) of SAN–TAPP and PP, which results in higher crosslinking density in the PP/SAN–TAPP composites, thereby leading to lower mobility of PP chains [42].

#### TG analysis

**Fig. 11:**
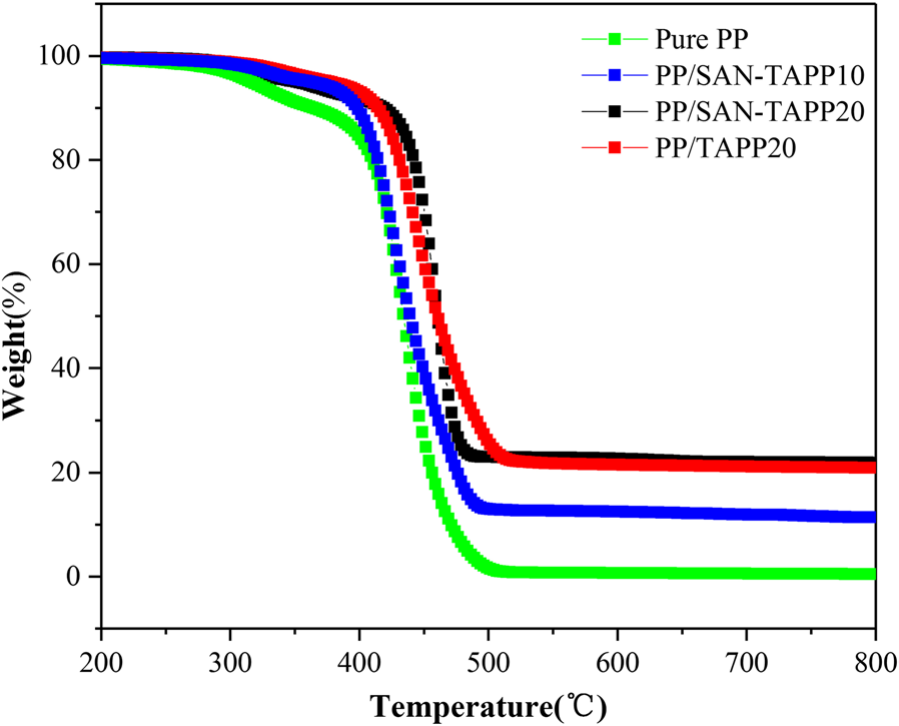
TG curves of pure PP, PP/TAPP20 and PP/SAN–TAPP composites

To assess the impact of SAN–TAPP on the thermal stability and thermal degradation behaviors of PP/SAN–TAPP composites, TGA analysis was carried out under N_2_. Figure 11 shows the TG curves of pure PP, PP/TAPP20 and PP/SAN–TAPP composites. The 10% weight loss temperature and maximum degradation loss temperature rate (T_max_) are shown in Table 2. Upon Fig. 11, pure PP started to decompose at 363.9 °C, and showed a one-stage degradation with a T_max_ of 438.9 °C. The macromolecular chain of pure PP was almost completely converted into volatile product with char residue of 0.48%. The 20 wt% TAPP filled PP/TAPP20 composite and PP/SAN–TAPP composites underwent a degradation apparently similar to pure PP. The 10% weight loss temperature of 20 wt% TAPP filled PP/TAPP20 composite and PP/SAN–TAPP composites shifted to a higher temperature in comparison with pure PP. Besides, the T_max_ of 20 wt% TAPP filled PP/TAPP20 composite and 20 wt% SAN–TAPP filled PP/SAN–TAPP composite increased, with 12.4 and 17.5 °C higher as compared to pure PP, respectively. This observation depicted the enhanced thermal stability by adding SAN–TAPP into PP matrix. The residual char from 20 wt% TAPP filled PP/TAPP20 composite and PP/SAN–TAPP composites at 700 °C increased significantly. PP/SAN–TAPP20 had a residual char of 20.8%, which was close to that of 20 wt% TAPP filled PP/TAPP20 composite. These results reveal that SAN–TAPP enhances the thermal stability and functioned as an effective thermal shield in PP matrix, which can be ascribed to the cooperative catalytic carbonization effect between the APP and SAN phase retards the escape of pyrolysis volatile and decrease the heat transfer and mass loss [31].

**Table 2:** Degradation temperature of pure PP, PP/TAPP20 and PP/SAN–TAPP composites

Samples	T (°C) at 10% weight loss	T (°C) at maximum weight loss
PP	363.9	438.9
PP/TAPP20	408.8	451.3
PP/SAN–TAPP5	367.8	442.6
PP/SAN–TAPP10	389.3	448.3
PP/SAN–TAPP15	397.0	451.2
PP/SAN–TAPP20	406.4	456.4

#### HDT analysis

**Fig. 12:**
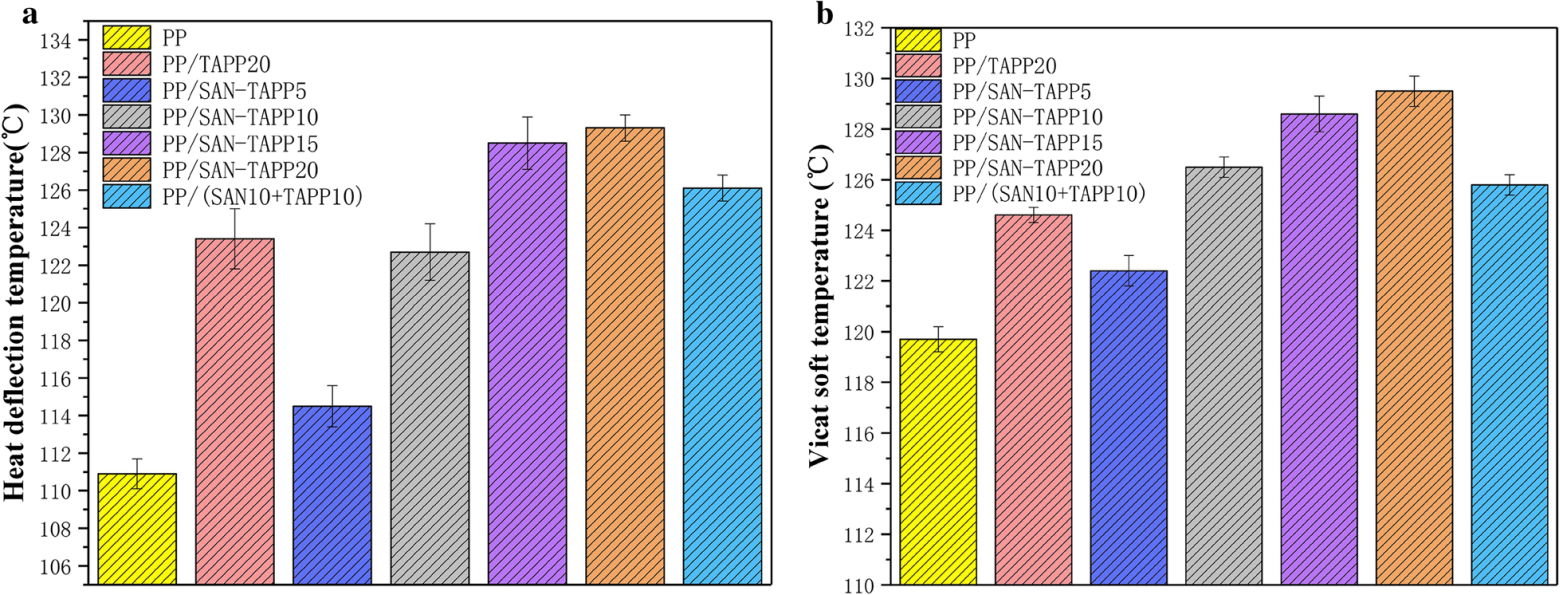
**a** Heat deflection temperature; **b** Vicat softening temperature of pure PP, PP/TAPP20 and PP/SAN–TAPP composites

The heat deflection temperature (HDT) is considered to be a parameter that can be used to measure a certain creep-compliance temperature after the material has been subjected to a standard temperature program and a certain weight loading [40]. Figure 12 shows the HDT and vicat softening temperature (VST) of pure PP, PP/TAPP20 and PP/SAN–TAPP composites. As shown in Fig. 12, PP/TAPP 20 and PP/SAN–TAPP composites presented an increase in HDT and VST temperatures, as compared with pure PP. Gradual increases of the HDT and VST value were observed as the SAN–TAPP content was increased. It is worthy to note that 20 wt% SAN–TAPP filled PP/SAN–TAPP composite had a higher HDT and VST value than that of PP/TAPP20 composite and 10 wt% SAN- and 10 wt% TAPP-filled PP/SAN–TAPP composite. It was likely attributed to 20 wt% SAN–TAPP filled PP/SAN–TAPP composite’s finer mechanical properties and higher thermal stabilities which were resulted from the more refined phase morphology and sea-island structure [19].

### Flammability properties of pure PP, PP/APP20 and PP/SAN–TAPP composites

The flame retardancy of pure PP, PP/APP20 and PP/SAN–TAPP composite was assessed by LOI and UL-94 tests. The data are summarized in Table 3. Pure PP is well known to be inflammable, with a LOI value of 19.8%. The 5 wt% SAN–TAPP filled PP/SAN–TAPP composite had a slight increased LOI value of 21.4%. As the content of SAN–TAPP increased, the LOI value gradually increased. With the addition of 20 wt%, the value was improved to 27.5%, which approached that of PP/TAPP20 composite and was higher than 10 wt% SAN- and 10 wt% TAPP-filled PP/SAN–TAPP composite. In terms of the UL-94 test, pure PP, 5 and 10 wt% SAN–TAPP filled PP/SAN–TAPP composites and 10 wt% SAN- and 10 wt% TAPP-filled PP/SAN–TAPP composite had no rating owing to their flaming time longer than 30 s and high or moderate dripping. The 15 and 20 wt% SAN–TAPP filled PP/SAN–TAPP composites and PP/TAPP20 composite reached V-2 rating. These results indicate that the SAN–TAPP could exert a cooperative catalyzing carbonization effect during combustion process, which is likely due to the encapsulation of SAN–TAPP and the chemical reaction between SAN and TAPP that would promote the formation of a carbonized romantic networks and cross-linked phosphorus oxynitride which retards the escape of pyrolysis volatile and inhibits the inner materials exposed to fire during combustion [43–45]. Nevertheless, further investigation is needed to determine the specific enhancing mechanism of the SAN–TAPP flammability. In future research, we hope to apply the enhanced polypropylene composite to a broader field of research, especially in the screening for active components of Chinese medicines [46, 47].

**Table 3:** The LOI and UL-94 values of pure PP, PP/TAPP20 and PP/SAN–TAPP composites

Samples	LOI (%)	UL-94	Remark
PP	19.8	No rating	High dripping
PP/SAN–TAPP5	21.4	No rating	High dripping
PP/SAN–TAPP10	23.3	No rating	Moderate dripping
PP/SAN–TAPP15	25.5	V-2	Low dripping
PP/SAN–TAPP20	27.5	V-2	Low dripping
PP/(SAN10 + TAPP10)	23.4	No rating	Moderate dripping
PP/TAPP20	27.8	V-2	Low dripping

## Conclusions

In our study, SAN resins encapsulated functional titanate-modified APP (TAPP) to produce a flame retardant for PP, SAN–TAPP, which is then added to PP composites to simultaneously improve their mechanical properties and thermal stability. The XRD result demonstrated that SAN–TAPP had no obvious effect on the crystal form of PP. According to SEM images, PP/SAN–TAPP composites had a sea-island morphology with irregular spheres and dark cavities. Impact strength of PP/SAN–TAPP composites was significantly improved, especially for15 wt% SAN–TAPP filled PP/SAN–TAPP composites. Their tensile strength and modulus were also higher than pure PP. The improvement in mechanical strength is most likely due to sea-island morphology and even dispersion of SAN–TAPP in the PP matrix. The DSC, TG and HDT results demonstrated that T_m_, HDT and residual char yield were increased by the addition of SAN–TAPP. Furthermore, the LOI value of the PP composites increased with addition of SAN–TAPP. The 15 and 20 wt% SAN–TAPP filled PP/SAN–TAPP composites passed the V-2 test of UL-94, and exerted the similar effect on the flame retardancy as TAPP with the same loading. The enhancement of thermal stabilities is probably due to the cooperative reinforcement effect of TAPP and SAN and the interfacial reaction (interaction) of SAN–TAPP and PP.
